# Social and economic variables explain COVID-19 diffusion in European regions

**DOI:** 10.1038/s41598-024-56267-z

**Published:** 2024-03-13

**Authors:** Christian Cancedda, Alessio Cappellato, Luigi Maninchedda, Leonardo Meacci, Sofia Peracchi, Claudia Salerni, Elena Baralis, Flavio Giobergia, Stefano Ceri

**Affiliations:** 1 Department of Control and Computer Engineering (DAUIN), Politecnico di Torino, Turin, Italy; 2 Department of Management, Economics and Industrial Engineering (DIG), Politecnico di Milano, Milan, Italy; 3 Department of Design (DESIGN), Politecnico di Milano, Milan, Italy; 4 Department of Electronics, Information and Bioengineering (DEIB), Politecnico di Milano, Milan, Italy

**Keywords:** Scientific data, Risk factors

## Abstract

At the beginning of 2020, Italy was the country with the highest number of COVID-19 cases, not only in Europe, but also in the rest of the world, and Lombardy was the most heavily hit region of Italy. The objective of this research is to understand which variables have determined the prevalence of cases in Lombardy and in other highly-affected European regions. We consider the first and second waves of the COVID-19 pandemic, using a set of 22 variables related to economy, population, healthcare and education. Regions with a high prevalence of cases are extracted by means of binary classifiers, then the most relevant variables for the classification are determined, and the robustness of the analysis is assessed. Our results show that the most meaningful features to identify high-prevalence regions include high number of hours spent in work environments, high life expectancy, and low number of people leaving from education and neither employed nor educated or trained.

## Introduction

At the end of 2019, the COVID-19 disease started spreading around the world. The first case of COVID-19 in Italy was confirmed in Lombardy on February 20, 2020. The first patient was admitted to a hospital in Codogno (Lombardy, Italy) with a “mild pneumonia resistant to therapy, no relevant travel history and no apparent exposure to diseased contacts”^1^. In the following week, the Codogno area saw a rapid increase of COVID-19 cases, as well as other areas in the southern part of Lombardy^1^. At the beginning of 2020, Italy was the nation with the highest number of cases among not only European countries, but the rest of the world^2^. Lombardy, with its 10 million inhabitants, was the most heavily hit region in Italy, with one third of all cases and half of all deaths^2^.

The main objective of this work is to identify the main variables that explain the spreading of the virus in the European regions. We collected variables belonging to six categories: education, demography, healthcare, mobility, primary sector, economy. Specific variables were selected on the basis of their availability on an adequate spatial scale. We characterized European regions at a sub-national granularity that has allowed to reach a good balance between the research goals and data availability.

The adopted methodology consists in using several machine learning algorithms to learn about the main associations between the collected variables and the risk of exposure of each European region (quantified by the density of COVID-19 cases registered during the first and second waves). Next, we analyze the machine learning models that have been trained to extract useful insights in terms of the importance that each model assigns to the various available features. Since we study both the first and the second COVID-19 waves, we additionally make comparisons between the role that the features played in both situations.

As proximity is key to viral diffusion^3^, several works study the spreading of COVID-19 in space and time. Focusing on Italy, De Angelis^4^ and Bontempi^5^ analyzed Lombardy data at the municipality level, while Coccia^6^ and Cartenì^7^ considered the whole Italian territory, either at the provincial level or at the regional one. Other studies addressed several European countries: Bontempi^8^ focused on Italy, Spain and France, while Kapitsinis^9^ included 9 European countries. For what concerns the temporal dimension, some works focused on the initial period of the first wave (between January and April 2020)^4–6,9^, other works concentrated on the second wave (between September and December 2020)^7^, and other works focused on discontinuous short intervals: for example, Bontempi^8^ focused on April 2020, November 2020 and January 2021, the peaks of COVID-19 diffusion in Europe. Such strategy of analysis of epidemiological phenomena has its roots in mathematical epidemiology^10–12^, in which the spread of the disease under study is modeled by means of susceptible-infected-recovered (in short SIR)^11,13^ models at a chosen geographic granularity, separately for each time-period based on the employment of pharmacological or non-pharmacological containment measures. The standard SIR model and its variations of increasing complexity(e.g. SIRV, SEIR, SEIRS) have already been applied at different geographic scales, from region^14^, to country^15,16^ and continent level^17,18^ in order to develop a thorough understanding on the risk of transmission of COVID-19. To this end, risk estimation of COVID-19 spread has been studied from the perspective of time-varying SIR models^19^. Nonetheless, following studies showed that SIR is an ineffective modeling strategy^20^ for the COVID-19 epidemic, as such technique does not account for the contributing factors which have been shown to be greatly correlated with the spread of COVID-19, such as pollution, social norms and cultural context of each population^20^.

For what concerns the choice of the factors which may have contributed to COVID-19 diffusion, some studies focused on environmental and meteorological data^5,21,22^, other studies focused on mobility: Sannigrahi^23^ analysed how the volume of international commerce influenced the spread of COVID-19, while Cartenì^7^ analyzed how public transport influenced the spreading of COVID-19 in the second wave. Differently from these works, we consider a larger set of European regions and contributing factors.

Several works take into consideration a variety of contributing factors. The most common macro areas analysed regard demography, economy or the healthcare system^23–26^, sometimes integrated with environmental^6^ or political factors^27^. Some studies argued that the strongest correlated factors with COVID-19 are socio-economic (population, poverty, income, GDP)^8,25,28^; other studies assessed a major correlation of environmental factors such as air quality^5,9^ or air pollution^22^. In other cases, spatial and geometrical properties (e.g. of cities) have been studied to understand their relationship with the spread of COVID-19^29^.

Some studies disagree, perhaps due to the difference in datasets and methods: for instance, Amdaoud^25^ argued that better hospitals and medical services were strongly correlated with lower mortality rates, but Lupu^24^ stated that they did not affect at all the number of deaths. Most of the studies agreed that factors such as life expectancy^9,25,26,30^ or population density^22,31^ are highly correlated both with mortality and number of COVID-19 cases. What is crucial to understand is how these factors correlate to other socio-economic and healthcare elements, in order to create a more encompassing model, that takes in consideration the various aspects of our society. Hence, this work has the objective to explore, from both a temporal and a geographical perspective, how these various socio-economic and healthcare elements have had an effect on the spread of COVID-19.

## Materials

The dataset used for this study includes a subset of European regions, as defined by the NUTS2 classification. The NUTS (Nomenclature of Territorial Units for Statistics) is a hierarchical system for dividing the economic territory of the European Union (EU)^32^. The NUTS nomenclature identifies regions at three different levels (NUTS 1, 2, 3), moving from larger to smaller territorial units. In particular, the second NUTS level (NUTS2) identifies regions within countries (for example, in Italy NUTS2 identifies regions such as Lombardy or Tuscany). As a general representation, each NUTS2 region is identified by a code that concatenates the NUTS1 identifier and an incremental region identifier. For example, Italian regions Lombardy and Tuscany are identified by *ITC4* and *ITI1* respectively. In this case, *ITC* and *ITI* are the NUTS1 representations for north-west and central Italy. A full list of all NUTS levels is openly available^32^.

The regions considered for this study belong to a total of 20 European countries. The total population living in these regions is of 357 million people.

For each of the regions under study we collected a total of 22 variables from the Eurostat data repositories. Eurostat is the statistical office of the European Union^33^. The variables span six macro areas that have been found in literature to be relevant in relation to the spread of viruses: economy, education, population, healthcare, primary sector and mobility.

Although the data is made available by Eurostat, this Office does not generally engage in direct data collection. Instead, data is gathered in individual European countries by their respective national statistical authorities, adhering to standardized European statistical regulations and standards, overseen by Eurostat. National authorities are responsible for verifying and analyzing their data, which is subsequently transmitted to Eurostat. The organization performs validation and quality control checks on the received data. These data sets are systematically published in accordance with a predefined publication calendar^34^.

We additionally gathered information on the impact that the first and second COVID-19 waves had on the various regions, based on data gathered from the Joint Research Centre Repository^35^. The data has been collected at the sub-national level and includes the numbers of infections by COVID-19, collected directly from the National Authoritative sources (more specifically, on the National monitoring websites)^36^. Following^37^, we characterized the first wave as having occurred between February 20 and August 20, 2020, and the second wave between August 20, 2020 and February 20, 2021.

For each pandemic wave, we studied the density of cases and deaths per hundred thousands inhabitants. The number of deaths is strongly dependent on the preparedness of the local health systems to react to and manage the virus effects. Instead, the number of cases allows for a more precise characterisation of the diffusion environment in the regions under analysis. Based on the goal of trying to understand how viruses spread and what factors might influence future waves (or similar pandemic events), we consider the number of cases occurred as being a more interesting target.

The rest of this section presents a better characterization of the data used, with information about the data cleaning performed, the variables considered and the discretization that has been applied to the problem.

### Data cleaning

After collecting the socio-economic variables and the COVID-19 outcomes, we applied an initial data cleaning step. From the pool of all available regions, Eurostat data were available only for 205 NUTS2 regions. Coronavirus data collected from the JRC repository were available only for 154 and 152 regions, for the first and second wave respectively. Thus, only regions for which both the covariates and coronavirus cases data were available could be considered. Furthermore, we note that the information on the population of a region is necessary to preprocess the data (e.g. compute the various densities). For this reason, 3 regions have been removed due to absence of this value. After applying all of these constraints, a total of 151 and 149 regions have been analyzed for the first and second wave.

Consequently, since Sweden did not adopt pandemic containment procedures, all its 8 regions were removed from the analysis: the dynamics that occurred there are distant from those of the other European regions, which are the main focus of this study.

Additional attention has been paid to 6 NUTS2 regions which had missing values for covariates which, even if filled with the aggregate national statistic (computed considering the regions in the dataset), would result in extreme outlier values (unlikely with respect to the sampled distribution of the respective missing feature) after normalization by their populations. Hence, the regions *BG32*, *BG42* (in Bulgaria), *ES63*, *ES64* (in Spain), *FI20* (in Finland) and *FRY5* (in France), which presented the above stated anomalous behaviors, have also been removed.

We thus obtained a dataset of 137 NUTS2 regions for the first COVID-19 wave, and 135 NUTS2 regions for the second one.

### Variables

The 22 variables collected from Eurostat can be divided into six different categories, which, according to literature, are relevant in their relation to the spread of viruses: education, population, healthcare, mobility, primary sector and economy.

- *Education* variables include information about early leavers from education and training, NEET rate (people Not engaged in Education, Employment or Training), students enrolled in education, students in tertiary education and participation rate in education and training.
- *Population* variables regard life expectancy, crude death rate, population density and total deaths.
- *Healthcare* variables considered for this study include those related to long-term care beds, hospital beds, hospital discharges for respiratory diseases and health personnel.
- *Mobility* variables cover the volumes of vehicles and air passengers.
- *Primary sector* variables consider data about farm labour force and utilized agricultural area.
- *Economic* variables include unemployment rate, thousands of hours worked, GDP (Gross Domestic Product), compensation per employee and regional GVA (Gross Value Added – the total value of all goods and services produced subtracted of the value of goods and services used for intermediate consumption in their production).

For each variable, we select the latest available measurement before the outset of the pandemic, representing the pre-pandemic condition. When meaningful, the variables have been typically normalized per 100,000 inhabitants. A full description of each variable is available in Table 1.

**Table 1:** Brief description for each of the 22 variables used for the study.

Category	Variable	Description	Unit of measure
Education﻿	Early leavers from education and training	Percentage of young people (between 18 and 24 years old) who left school, university or training classes after having started them, over the total number of people who joined schools, universities and training	Percentage
	Students enrolled in tertiary education	Total number of students (independent from sex and age) enrolled in tertiary school education	Absolute number
	NEET rate	Percentage of people from 15 to 24 who are Not engaged in Education, Employment or Training (NEET) over the total number of people of that age	Percentage
	Participation in education and training	Percentage of people between 25 and 64 years old who, in the last 4 weeks, has participated in educational and training activities	Percentage
	Pupils and students enrolled	Total number of students (independent from sex and age) enrolled in school	Absolute number
Population	Life expectancy	Life expectancy for a person	Years
	Population density	Density of population in each region	People/$km^2$
	Crude death rate	Mortality in relation to the total population, Expressed in deaths per 100,000 inhabitants	Per 100,000 population
	Deaths	Number of deaths in each region	Absolute number
Healthcare	Hospital discharges for respiratory diseases	Number of people who left the hospital after having suffered from respiratory diseases	Absolute number
	Long-term care beds	Number of long term beds available in a region for every 100,000 inhabitants	Per 100,000 population
	Health personnel	Health care staff, the “manpower” active in the health care sector (e.g. doctors, dentists, nurses)	Absolute number
	Available hospital beds	Number of available hospital beds in a region, expressed as average per 100,000 inhabitants	Per 100,000 population
Mobility	Air passengers	Number of passengers carried, in that region, per thousand of inhabitants	Per 1,000 population
	Stock of vehicles	Total number of vehicles present in a region	Absolute number
Primary sector	Farm labour force	Regular labor force involved in farm work. Expressed in Annual Work Units (AWU), where 1 AWU corresponds to the work performed in a year by one full-time person	AWU
	Utilized agricultural area	Share of the utilizied agricultural area (UAA) occupied by the main agricultural land uses (arable land, permanent grassland and land under permanent crops)	Percentage
Economy	Unemployment rate	Unemployment rate in a region	Percentage
	Thousands of hours worked	Sum of the hours worked by employees in a certain area	Absolute number
	Real growth rate of regional GVA	GVA (Gross Value Added) is an indicator of the economic activity of a country or region. It reflects the total value of all goods and services produced minus the value of goods and services used for intermediate consumption in their production	Percentage change w.r.t. previous period
	Compensation of employees	Sum of the compensations of all employees of a certain area	Millions of euros
	GDP	Gross Domestic Product (GDP) at current market prices	Millions of euros

**Figure 1:**
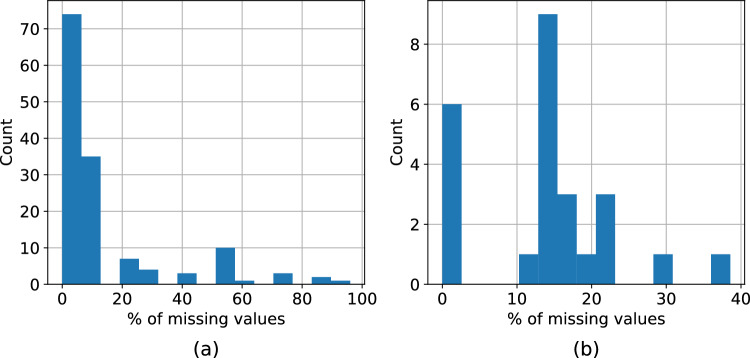
Analysis of missing values in the adopted dataset. (**a**) shows the distribution of the fraction of missing features for each region. (**b**) shows the distribution of the fraction of missing regions for each feature.

The dataset collected in this way contains some missing values. Figure 1a and b show the distribution of missing variables for each region and the distribution of missing regions for each variable, respectively. The region with the majority of missing variables is “Northern and Eastern Finland”, with all but one feature missing. The feature with the highest number of missing values is instead “longterm care beds”, whose information is missing from 54 regions ($\approx$ 39% of regions). The missing values have been filled by using each region’s national average for the selected feature.

### Risk classes of COVID-19 diffusion

We are interested in building classes of risk for the various regions, according to their risk of exposure in terms of COVID-19 cases. This helps define a clearer narrative for the general public: because of this, several countries have already adopted similar risk-based classes to provide different guidelines and enforce different policies (e.g. in Italy, yellow, orange and red zones have been defined based on each region’s risk).

**Figure 2:**
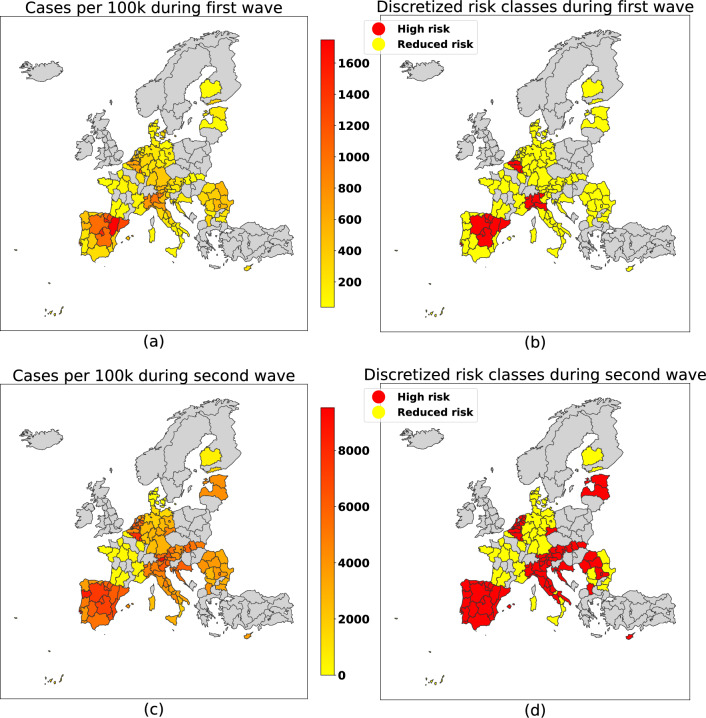
ROC curves for the different predictive models with respect to the first (left) and second (right) waves. Curves close to the (0, 1) corner achieve better performance. (**a**) shows the ROC curve for the first wave: the random forest generally achieves better performance. (**b**) shows the ROC curve for the second wave: in this case, all models obtain similar results. All results have been obtained using leave-one-out validation. The positive class is considered to be the high risk one. The blue dotted line represents random guess.

We build risk classes according to each region’s risk of exposure to COVID-19 cases by performing a 1-dimensional k-means^38^ unsupervised clustering algorithm on the number of cases for each wave, with a varying number of clusters: we found that two clusters is an optimal choice, in terms of silhouette score^39,40^ and of numerosity of each risk class. To obtain a more robust choice of clusters, we used multiple centroids initialization and selected the cluster assignment with the lowest sum of square distances from the points to their cluster’s centroids. It should be noted that applying k-means to a 1D dataset has the same effect as defining $k-1$ thresholds to use to group the data points. We still used k-means instead of manually defining the thresholds both to obtain a meaningful value for *k* (from a *k*-vs-silhouette plot) and to automate the selection of the threshold value. The COVID-19 cases density and the resulting discretized risk classes are separately shown for the two waves in Fig. 2.

## Methods

The objective of this work is to identify and quantify the effect that various socio-economic variables have in determining the risk class associated with each European region. To achieve this goal we opted for a classification-based machine learning approach, using models that provide some kind of interpretation of the results obtained. The machine learning pipeline adopted is comprised of the following steps: minority class rebalancing, model training, tuning and evaluation; followed by a final interpretation of the results. The rest of this section presents each of these steps in additional detail.

All experiments have been performed using a machine running Ubuntu 20.04, equipped with an Intel Xeon X5650 (6 cores, 12 threads) @ 2.66 GHz and 32 GB of memory. The source code has been developed using Python 3.8 and the scikit-learn^42^ library for the main proposed models. A link to the source code repository is made available as a part of the Data availability statement.

### Minority class rebalancing

The two classes (high and low risk) are not well-balanced for the two waves. For this reason, we use SMOTE^43^ (Synthetic Minority Over-sampling Technique), a commonly adopted technique to rebalance the problem. We have found the best performance in terms of downstream classification task for a number of neighbors for the interpolation $N = 6$. This choice is found to be a stable one, since we also achieve similar performance for other similar values of *N*.

### Model training and tuning

We trained various classifiers that can be inspected to extract meaningful considerations on the importance of the input features. Hence, we used a subset of interpretable methods from the statistical learning literature, namely: *logistic regression* (LR), *Support Vector Machine* (SVM)^44^ with a linear kernel and *random forest*^45^ (RF). Logistic regression allows to infer from the available data, the relationship that exists between the features of an input sample and the respective predicted class, based on the odds of membership to one class with respect to all others.

Random Forest is an ensemble learning method which achieves similar goals by means of majority vote prediction from multiple separately trained decision trees, each fit on a subset of features(features bagging).

The Support Vector Machine instead solves the classification problem by finding the hyper-plane that best separates the data points according to the respective classes. This fact is then used to predict the class associated with a test sample, based on the side of the found plane it is located.

We are mainly interested in studying the “high risk” class, i.e. the most alarming of the two classes. Because of this, we measure precision and recall of all classifiers obtained for the high risk class. To find a balanced model, we additionally consider the $F_1$ score (i.e. the harmonic mean between precision and recall). We use this metric for the choice of hyperparameters and overall evaluation because both minimizing the false positives and false negatives is important for the purpose of our classification problem: a large number of false positives implies over-estimating the importance of features that are not indicative of high risk, whereas high false negatives would result in under-estimating the features that might instead be relevant for the high risk class.

To find the optimal hyperparameters we adopted a 5-fold cross validation technique, choosing for each model the configuration that maximizes the average $F_1$ score on the high risk class, across all folds. The following are the hyperparameters that have been considered for each model.

For the logistic regression, we considered using either an L1 or an L2 regularization term (with regularization coefficient $\in \{0.1, 0.5, 1, 5, 10, 20, 50\}$, as well as no regularization at all. For the random forest we trained models using 100 trees and a maximum depth that ranged from 2 to 10, as well as not enforcing any depth regulation. For the SVM we adopted a linear kernel (to obtain weights that can be easily interpreted), with a regularization parameter $\in \{0.1, 0.5, 1, 5, 10, 20, 50\}$. We also tune the class weight assigned to the low and high risk categories; for this parameter, the following combinations are selected (notation is *high risk weight* : *low risk weight*): $\{1 : 1, 4 : 1, 8 : 1, 32 : 1, \frac{\#samples}{\#classes\cdot \#samples_{high\_risk}}~:~\frac{\#samples}{\#classes\cdot \#samples_{low\_risk}}\}$.

### Model evaluation

The models are evaluated by computing the leave-one-out $F_1$ score on the dataset of each wave. The leave-one-out validation strategy has not been used during the hyperparameter tuning phase because of its computational complexity. However, it provides a good estimate of the model’s performance on new data points.

As the tuning choices have been made on the dataset through k-fold cross validation, the overall performance metrics reported for leave-one-out are not independent of the performance obtained during validation. We are aware of this, but still opted for this evaluation technique due to the limited data availability – which would only allow for a small test set to be set aside.

### Feature selection and interpretation

Three ML methods (logistic regression, linear SVM, random forests) have been used for feature selection. Each model has been trained with its best hyperparameter configuration and used to establish the relationships between the 22 variables and the risk class prediction. Each model has its means to quantify the relevance of the relationship between a variable and the risk classes, according to the available data. For both linear SVM and logistic regression, each of the parameters of the models provides a measure of the degree by which each feature skews the likelihood that a test sample should be classified with a given label or not.

Hence, the risk class prediction is the result of the weighted contribution of each variable of the test sample. In the case of Random Forest instead, a measure of absolute relevance, namely the feature importance, is attributed to each variable as a means to quantify its impact in the class prediction procedure. Although it is not directly employed for inference by the model (as is the case of the coefficient of LR and SVM), it allows to measure the influence of each variable in the overall majority vote class prediction. Next, we discuss in more detail the interpretation of each algorithm.

**Logistic regression** provides the means to both classify regions and estimate the influence of each feature on the odds of the risk class^46^ of any given NUTS2 region. The optimization objective defined below allows us to find the set of coefficients $W \in {\mathbb {R}}^d$ which define the relevance of all features for the purpose of classification. Here, $x_i \in {\mathbb {R}}^d$ represents the set of features of the *i*-th sample of our dataset.

$$\begin{aligned} \min _{W \in {\mathbb {R}}^d, b \in {\mathbb {R}} } C \sum ^N_i \log (1+ e^{-y_i (W^T x_i + b ) } ) + \frac{1}{2} ||W||^2_2 \end{aligned}$$

In the case of L1 penalty term, the $\frac{1}{2} ||W||^2_2$ is substituted by the L1 norm $||W||_1$. For the logistic regression, the quantity $W^Tx_i + b$ represents the logarithm of the predicted odds ratio. As such, the coefficients in *W* have a direct relationship with the predicted probability of the positive class (high risk).

Similar considerations hold for **linear SVM**, which separates samples of the two risk classes by determining an hyperplane that maximizes the margin between two classes. Such separation frontier is defined by a set of coefficients obtained through an optimization routine: their values determine the relationship between each feature and the risk classes^47^. In the linear case, the optimization objective used to determine the optimal set of coefficients $W \in {\mathbb {R}}^d$ is:

$$\begin{aligned} \min _{W \in {\mathbb {R}}^d, b \in {\mathbb {R}} } C \sum ^N_i \max (0,1 -y_i (W^T x_i + b) ) + \frac{1}{2}||W||^2_2 \end{aligned}$$

Both of these models provide an indication of the direct or inverse influence that the features have on the identification of the low or high risk class.

Finally, **random forests** can be used to extract the importance that each feature has for the classifier^48^. This feature importance can be computed for each decision tree that comprises the random forest: this information can then be aggregated to compute an overall feature importance.

At any node of a decision tree, we can identify the set of samples to be processed as *D* and the two splits produced as $D_L$ and $D_R$, such that $D = D_L \cup D_R$ and $D_L \cap D_R = \emptyset$. For any of these sets of points we can compute an impurity value, which represents how “impure” they are with respect to the target class. We refer to this impurity score as $\iota (D)$. In this paper, we will discuss the impurity in terms of Gini index. The contribution of the split towards the overall separation of the classes in the dataset can be quantified as:

$$\begin{aligned} C(D) = |D|\iota (D) - |D_L|\iota (D_L) - |D_R|\iota (D_R) \end{aligned}$$

denoting the decrease in impurity achieved when passing from the parent to the children. Each impurity is weighted by the number of samples contained within the respective split. For each split, there is exactly one feature that is used for that split. As such, the contribution of the split can be assigned to the corresponding feature. It follows that for each feature *f* we can build a set ${\mathcal {D}}_f$ of all nodes that use that feature for the split. The overall contribution of feature *f* (or its feature importance), for tree *T* is then computed as:

$$\begin{aligned} fi_T(f) = \frac{\sum \limits _{ D \in {\mathcal {D}}_f} C(D)}{\sum \limits _{\phi \in F} \sum \limits _{D \in {\mathcal {D}}_\phi }C(D)} \end{aligned}$$

Where *F* is the set of all available features. The term at the denominator is used to normalize the result so that the sum of all feature importances is 1. For a random forest, the overall feature importance of any feature is given by the mean feature importance for that feature across all the trees ${\mathcal {T}}$. Once again, the feature importances obtained in this way are normalized so as to sum to 1.

$$\begin{aligned} fi(f) = \frac{\sum \limits _{T \in {\mathcal {T}}} fi_T(f)}{\sum \limits _{\phi \in F}\sum \limits _{T \in {\mathcal {T}}} fi_T(\phi )} \end{aligned}$$

## Results

In this section we present the results achieved for the models built, in terms of both performance and notions learned.

### Models performance

**Figure 3:**
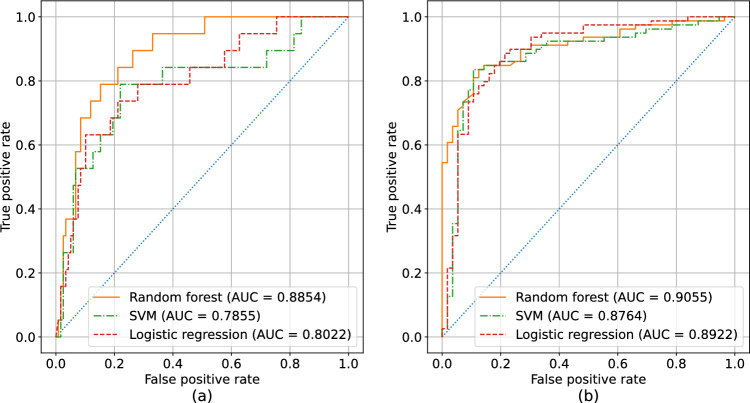
Coefficients learned by the linear SVM model for the first and second wave. Positive coefficients imply a positive weight of the respective feature toward the prediction of the “high risk” class, whereas negative coefficients characterize features more related to the “reduced risk” class. The magnitude of the coefficients is proportional to how impactful the respective feature is in the prediction. The coefficients are sorted by descending importance (in absolute value). (**a**) shows the coefficients learned by the linear SVM for the first wave, (**b**) shows the coefficients for the second wave.

In Table 2 we include the performance for both waves in terms of $F_1$ score, precision and recall on the “high risk” class. The first conclusion that can be drawn from this data is that all models have better predictive capabilities for the second wave – additionally all models are more consistent in terms of results achieved. Multiple reasons may be the cause of this: among them, we note that there has generally been a more accurate counting of COVID-19 infections during the second wave. This is the result of a general better preparedness w.r.t. the one shown by most countries for the first wave. The ROC (Receiver Operating Characteristic) curves for the best performing models are shown in Figure 3. Once again we observe a general consistency for the second wave, and an improved performance achieved by the random forest for the first wave.

**Table 2:** Performance of different predictive models with respect to both waves.

	First wave			Second wave		
	$F_1$ score	Precision	Recall	$F_1$ score	Precision	Recall
Logistic regression	0.5217	0.4444	0.6316	**0**.**8659**	0.8353	**0**.**8987**
SVM	0.4706	0.375	0.6316	0.859	**0**.**8701**	0.8481
Random forest	**0**.**5957**	**0**.**5**	**0**.**7368**	0.8447	0.8293	0.8608

All metrics are computed on the “high risk” class, using leave-one-out validation. In bold are the best performing models for each wave and metric.

### Predictive variables

We are mainly interested in extracting the importance assigned by each classifier to the variables under study. Since the models are characterized by different learning mechanisms, we first try to assess whether the models are consistent with one another in terms of importance assigned to the various variables under study.

To quantify the consensus of the classifiers in terms of the relevance of each feature, we first compute the Pearson correlation among the importances learned by each model. The results are shown in Table 3. To compare the feature importance of random forests (positive, larger for more important features) with that of SVM and logistic regression (positive or negative, larger in absolute value for more important features), the absolute value of the weights of SVM and logistic regression have been used.

**Table 3:** Correlation coefficient between each model’s feature importances.

		First wave			Second wave		
		LR	SVM	RF	LR	SVM	RF
First wave	LR	–	0.8917	ns	0.8423	0.5919	ns
	SVM	0.8917	–	0.5065	0.5876	0.4938	ns
	RF	ns	0.5065	–	ns	ns	0.4585
Second wave	LR	0.8423	0.5876	ns	–	0.7834	ns
	SVM	0.5919	0.4938	ns	0.7834	–	ns
	RF	ns	ns	0.4585	ns	ns	–

Feature importances for SVM and logistic regression are obtained as the absolute value of the learned weights. Cells containing “ns” represent situations when the correlation between models is not significant (p-value $\ge$ 0.05).

It can be observed that SVM and logistic regression make similar choices in terms of feature weighting, as denoted by large correlation values. An interesting fact is that, across the two waves, all models maintain a large positive correlation. This denotes that, although the two waves present many differences (as discussed next), all models make reasonably consistent choices in the selection of the relevance of the features.

**Figure 4:**
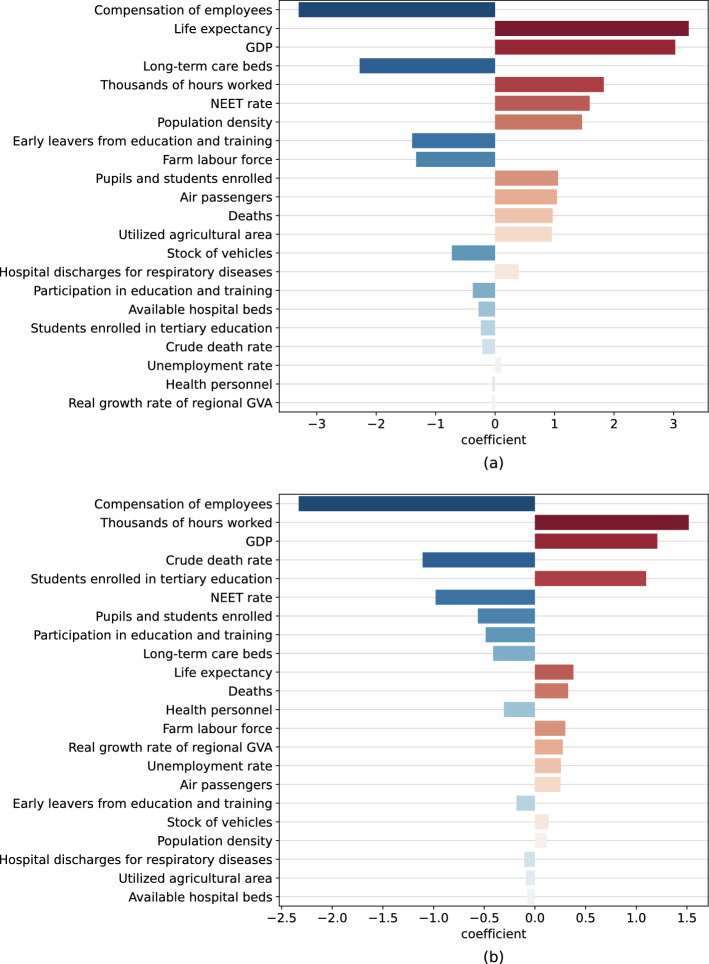
Feature distribution ordered from top to bottom by increasing p-value for an F-test applied on the data of the first (left) and second (right) waves. A lower p-value represents a more significant difference in means between the distributions of values between the high and reduced risk classes. A larger divergence may be leveraged by the classification models to identify differences between the two classes. All features have been standardized. (**a**) shows the distributions for the first wave, (**b**) shows the distribution for the second wave.

From Table 3 we can infer that the SVM has large correlation with both the random forest and the logistic regression. Because of this, we further analyze the coefficients learned by this model. These weights are shown in Fig. 4, separately for the first and second waves.

**Figure 5:**
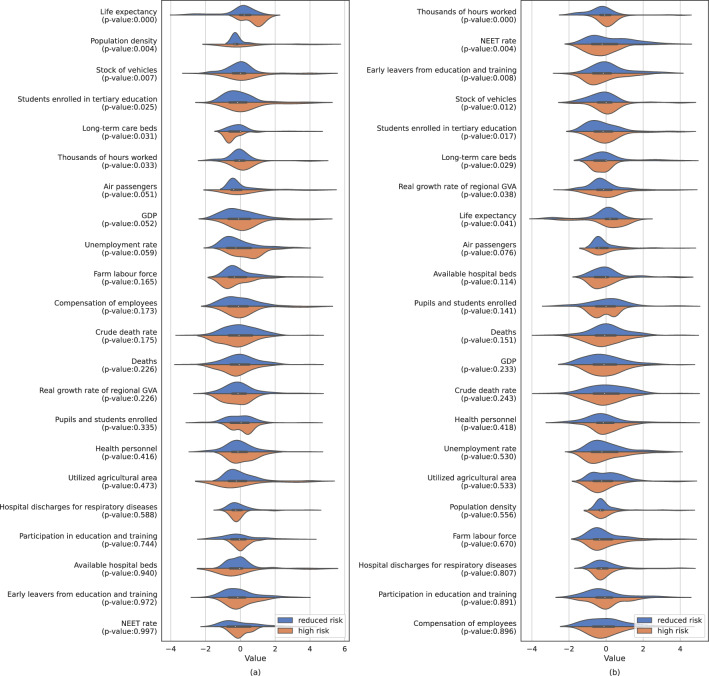
Side-by-side comparison of the continuous density of reported COVID-19 cases normalized per 100,000 inhabitants (left) and of the resulting discretization in binary severity classes (right), separately for first (top) and second (bottom) waves. Data obtained form the COVID-19 cases^35^. Borders have been defined using the NUTS2 GeoJSON definitions^41^. Python 3.8, GeoPandas 0.8 and Matplotlib 3.7 have been used to produce the images.

For a more expressive representation of the distribution of values, Fig. 5 shows the violin plots, separately for high and reduced risks, for both waves. The difference in distribution of values between the various features is the main drive of the magnitude of the weights learned. In other words, the more the distributions for a specific feature diverge, the larger the associated weight learned by the classification model will be.

#### The first wave

Considering the order of decreasing relevance from top to bottom of the graph, it can be observed that the economic variables such as compensation of employees, GDP and number of hours worked are some of the features which mainly affect the odds of a NUTS2 region to be at high risk of exposure to COVID-19 spread.

The number of hours worked and the population density are also relevant predictors which increase the odds of a region to be at high risk. On the other hand, employee compensation is inversely correlated with the risk classes.

Next, information about healthcare and demographic features have a considerable influence for the first wave. In particular, a high life expectancy and a high population density can be seen to have a positive effect on the prediction of a high risk class. The number of long-term care beds is instead negatively correlated with it. Although no causality can be inferred, we can hypothesise that an aging population has been affected more substantially by the first COVID-19 wave, that more densely populated areas can result in more widely spread infections, whereas a strong healthcare system can be assumed to have been useful to prevent many cases from further spreading.

#### The second wave

Similarly to the first wave, we observe that economic and socio-demographic indicators provide the greatest contributions to the classification models, together with those related to education; all the others only marginally improve odds in favor of either the low or the high risk class.

More specifically, the GDP and the number of hours worked have a positive impact on the prediction of the high risk class, whereas employee compensation is once again inversely proportional to the odds of being at high risk.

It is peculiar that life expectancy becomes much less impactful, while the crude death rate, the number of enrolled students in primary and in tertiary education now become relevant for classification.

While in general the most predictive features are consistent across waves, there are some cases that can be observed where inconsistent behaviors can be observed: most notably the NEET rate and the number of students enrolled in primary education change from positively to negatively correlated. Furthermore, the number of students in tertiary education changes from uncorrelated to highly positively correlated in the second wave. We can in this case advance the hypothesis that this change in effect for education variables can be explained in terms of the different roles that education has played in the two waves: the first wave has been characterized by students being prevented from attending schools and universities, whereas the second wave has often seen students being allowed to resume in-presence activities.

## Discussion

Both the first and second wave are characterized by a high similarity of the selected most relevant features. Socio-economic variables are ranked as highly relevant, with compensation of employees deemed to be the most influential factor with negative correlation to the high risk of the exposure to COVID-19 spread. The number of hours worked and GDP have also been determined as important, with positive proportional relationship to the increase in number of infections. This result is aligned to other studies that stated that the strongest correlation with the virus has to be associated to socio-economic metrics^5^, mainly in relation to the quality of life and the GDP^25,28^.

These factors are usually associated with modern developed economies, high ratio of urban population, the people’s inclusion in the service industry, high health system maturity and solid international migrant stocks. Thanks to this result it is possible to state that a high level of economic wellness is associated with a higher possibility to fight against the virus but, at the same time, it increases the possibilities for COVID-19 to spread, because the community is more dynamic and people interact more with each other. Indeed, the impact of the dynamicity of NUTS2 regions on the spread of COVID-19 can be observed by the change in relevance of some features that appear as highly relevant in one wave but not on the other.

Such distinctive characteristic is remarked by the increase in relevance of the number of hours worked, from fifth to second most relevant feature for risk classification during the second wave, as well as the major increase in importance of the number of students in primary education(represented by “students enrolled”) and tertiary education (“students tertiary education”). Both are only slightly correlated to the risk classes in the first wave, while instead they become negatively and positively correlated respectively, during the second wave. On the other hand life expectancy, population density and number of long-term care beds are shown as highly influential only for classification on the first wave.

The negative correlation shown by the latter is supported also by the documented struggle of the European healthcare systems in managing the number of COVID-19 cases during the first months of the pandemic, especially if patients were to manifest severe symptoms^49^. Not all the patients affected by the virus were automatically admitted to local hospitals, due to the shortage of available beds and rooms, and since from March to May 2020 the majority of European citizens were in full-lockdown^50^, ill people were more likely to infect the other people living with them, therefore leading to a growth in the number of cases. As already shown in previous works^9,25,26^, life expectancy, strongly correlated with demographic factors such as number of elders within a population or quality of life in a specific area, is strongly linked to the number of COVID-19 cases (as well as deaths caused by the virus).

Moreover, given the particular lockdown conditions imposed during the first wave of the epidemic, population density and the NEET rate showed as crucial factors, because the higher the number of citizens, the easier COVID-19 transmission becomes, in both lockdown and non-restricted contexts^22,31^, since nations’ ability to maintain safe distances between citizens is jeopardized^51^. It is necessary to note that although lockdowns were imposed as soon as the virus had been detected in Europe in late February, the high positive correlation of the thousand hours worked, hence time spent in office environments, can only be explained by the research of Ref.^52,53^ that provides evidence for the presence of COVID-19 in Europe as early as January of 2020 or the last months of 2019, as social dynamics had been limited soon after February 2020.

As demographics and healthcare related features partially characterize the first wave of COVID-19, socio-economic variables become predominant for the second wave. The major importance of socio-economic dynamics for the second wave is shown by features such as the thousand hours worked, compensation of employees, NEET rate, which proxy the characteristics of the working population and the ones associated to the demographics of primary and tertiary education. It can be observed that crude death rate becomes relevant, with negative proportionality relationship, during the second wave. The case of the NEET (Not in Education, Employment, or Training) rate is rather peculiar, as it changes from positively to negatively correlated, to the odds of high risk of COVID-19 spread from the first to the second wave. As this slice of the total population is not an active part of the workforce, a greater NEET rate is shown to be associated to lower risk of the spread of COVID-19 during the second wave, while it is instead positively correlated for the first wave, in which, to most of the workforce was imposed smart-working modality. Thus, COVID-19 infection was facilitated regardless of the employment status.

This might also suggest that countries have provided a more adequate response, from the healthcare standpoint, in managing the ongoing pandemic during the second wave. In spite of this, the partial return to on-site working modalities during the second period of the epidemic resulted in an increase of COVID-19 diffusion due to the increase in human-to-human interactions that characterizes the professional world.

## Conclusions

This study analyzed the impact of various social and economic factors on the spread of COVID-19 across European regions during the first and second waves of the pandemic. Machine learning models were able to identify key variables that influenced the risk classification of regions into high or low prevalence of cases. For both waves, socio-economic variables such as hours worked, GDP, and unemployment proved most important. However, the influence of specific factors differed between waves. For the first wave, demographic traits like life expectancy and healthcare capacity also played a role. For the second wave, education levels and crude death rates became more predictive. This suggests countries adapted their responses over time. Overall, our results provide insight into how regional characteristics combined with containment measures shaped COVID-19 transmission dynamics across Europe.

There are several avenues for continuing and expanding this research. First, additional waves of data could be analyzed to see how predictive factors evolve as the pandemic progresses. Demographic and behavioral changes over time may influence transmission risks. Second, incorporating mobility and social contact network data could yield a more granular understanding of spread. Third, performing similar analyses at finer geographic scales, such as the municipality level, may reveal localized transmission patterns.

## Data Availability

The source code and the data used for this study are openly available at https://github.com/Chris1nexus/covid-eu-analysis.

## Abbreviations

AUC: Area under the curve
AWU: Annual work units
COVID-19: Coronavirus disease 2019
EU: European Union
GDP: Gross domestic product
GVA: Gross value added
JRC: Joint research centre
LR: Logistic regression
NEET: Not engaged in education, employment or training
NUTS: Nomenclature of territorial units for statistics
NUTS2: Nomenclature of territorial units for statistics, level 2
RF: Random forest
ROC curve: Receiver operating characteristic curve
SMOTE: Synthetic minority over-sampling technique
SVM: Support vector machine
UAA: Utilized agricultural area
