# Publisher Correction: Social and economic variables explain COVID-19 diffusion in European regions

**DOI:** 10.1038/s41598-024-58218-0

**Published:** 2024-03-28

**Authors:** Christian Cancedda, Alessio Cappellato, Luigi Maninchedda, Leonardo Meacci, Sofia Peracchi, Claudia Salerni, Elena Baralis, Flavio Giobergia, Stefano Ceri

**Affiliations:** 1 Department of Control and Computer Engineering (DAUIN), Politecnico di Torino, Turin, Italy; 2 Department of Management, Economics and Industrial Engineering (DIG), Politecnico di Milano, Milan, Italy; 3 Department of Design (DESIGN), Politecnico di Milano, Milan, Italy; 4 Department of Electronics, Information and Bioengineering (DEIB), Politecnico di Milano, Milan, Italy

Correction to: *Scientific Reports* 10.1038/s41598-024-56267-z, published online 13 March 2024

The original version of this Article contained errors in Affiliations 2, 3 and 4 respectively, where the city was incorrectly given as ‘Turin’. The correct affiliations are listed below.

2 Department of Management, Economics and Industrial Engineering (DIG), Politecnico di Milano, Milan, Italy.

3 Department of Design (DESIGN), Politecnico di Milano, Milan, Italy.

4 Department of Electronics, Information and Bioengineering (DEIB), Politecnico di Milano, Milan, Italy.

The original Article has been corrected.

